# Two Interesting Forms of Lameness

**Published:** 1898-01

**Authors:** W. E. A. Wyman

**Affiliations:** M’killip Veterinary College—Pro Tempore, Clemson College, S. C.


### TWO INTERESTING FORMS OF LAMENESS.

BY W. E. A. WYMAN, V.S.,
M'KILLIP VETERINARY COLLEGE—PRO TEMPORE, CLEMSON COLLEGE, S. C.

AMONG the cases exhibited at the Post-Graduate Clinic of the McKillip Veterinary College, the following two cases of lameness are of especial interest:

CASE I. *Disease of the head of the suspensory ligament* (*hind-leg*).—*History:* Over-exertion, traumatism, especially apt to occur in spirited animals, pullers, etc.; one or both legs may be involved.

*Inspection:* In the standing posture, all four legs are brought toward the centre of gravity. The fetlock of the affected leg shows more or less dorsal flexion. In very lame animals the lumbar region is arched, and the abdominal muscles are contracted. When walking and trotting a well-marked, swinging-leg lameness is seen. As the animal trots toward the observer the stifle appears prominent and the legs bowed. When trotting or walking by the observer an imperfect flexion of the hock, a shortened forward stride, and at the moment the function of the supporting leg begins, that is as the animal plants the foot, the diagnostic excessive dorsal flexion of the metacarpo-phalangeal articulation becomes apparent.

*Palpation:* In the early stages nothing, or a badly-defined soreness, can be detected, later on a secondary periostitis and its characteristic symptoms appear.

CASE II. *Paresis of the flexor pedis perforans and perforatus (hind-leg).—History:* Falls and slipping; animals which become cast in the stable and struggle violently. The animal never recovers fully.

*Inspection:* While standing still or backing, nothing abnormal is manifest. As soon as the animal walks, trotting is impossible, especially upon slippery ground; the hock is flexed excessively, the foot unduly advanced, all parts from the fetlock down are pendulous, plainly noticeable as the function of the swinging leg is about to cease. Just as soon as the function of the supporting leg begins the heels are put down first, and at this moment the hoof slides back from two to fourteen inches, depending upon the severity of the attack, to be repeated at each step. This peculiar action improves greatly when the animal is changed from soft footing to rough ground allowing a good foothold. Cases of long standing only show a limited retraction as the function of the supporting leg sets in.

*Palpation:* Nothing abnormal.

In describing the various forms of lameness, the writer considers such a division as the history of the case, its inspection and palpation, more practicable from a clinical standpoint.

The total absence of a work on the clinical diagnosis of lameness of the horse, a subject of vital importance to all surgeons, may serve as an apology for the creation of a work dealing fully with this subject and to be submitted to the profession at an early date.

